# The Impact of Peer-Based Recovery Support Services: Mediating Factors of Client Outcomes

**DOI:** 10.1007/s11414-024-09929-9

**Published:** 2025-01-14

**Authors:** Esther Quiroz Santos, L. A. R. Stein, Amy Stamates, Hailey Voyer

**Affiliations:** 1 Department of Psychology, University of Rhode Island, 130 Flagg Road, Kingston, RI 02881 USA; 2 Rhode Island Department of Behavioral Healthcare, Developmental Disabilities & Hospitals, Cranston, RI USA; 3 Department of Behavioral & Social Sciences and Center for Alcohol & Addiction Studies, Brown University School of Public Health, Providence, RI USA

## Abstract

Research demonstrates a positive impact of Peer Based Recovery Support Services (PBRSS) facilitated by peer recovery specialists (PRS), who are people in recovery from behavioral health conditions (e.g., substance use disorders [SUD] and mental health conditions). This study investigated PBRSS, their impact on client outcomes (e.g., substance use, health), and the factors (e.g., self-efficacy, perceived relationship with/helpfulness of PRS) mediating the relationship between services and outcomes while controlling for sociodemographic information (e.g., age). Data were collected across 58 sites within 25 agencies providing PBRSS in a state located in Northeastern USA. Cross-lagged panel models were used to examine 12 longitudinal mediational models in a sample of *N* = 412. Models were examined over two time periods (i.e., T1 and T2). After alpha correction (*p* = .00417), most results were nonsignificant. However, several findings indicated that constructs were significantly related across time in all models (e.g., self-efficacy at T1 significantly predicted self-efficacy at T2), while many point-in-time associations were also significant (e.g., number of services received was positively related to relationship/helpfulness of PRS at T1 and T2). Better PRS relationship/helpfulness at T1 significantly predicted a lower number of services received at T2, while receiving more services at T1 significantly predicted better PRS relationship/helpfulness at T2. Being older significantly predicted a worse overall health at T2 in some models. While no mediation was found, this study is important as it assists in building models with respect to the mechanisms by which PRS may effect change or not.

## Introduction

Substance misuse and its associated physical and mental health problems have become a major clinical and public health crisis in the USA.^1^ To address substance use and associated problems (e.g., homelessness, mental health), it is critical to identify potentially effective interventions and programming. In Peer Based Recovery Support Services (PBRSS), peer recovery specialists (PRS) are people in recovery from behavioral health conditions, which includes substance use disorders (SUD) and mental health conditions. PRS offer social support to others who are seeking to establish or maintain their own recovery,^2^ often through a harm reduction approach.^3,4^ Research evidence demonstrates a positive impact of PBRSS across treatment settings and clinical presentations.^5–7^ However, several limitations have been found in the literature evaluating PBRSS, including the role of peers being poorly defined, mixed findings in the literature, and methodological confines (e.g., poor control of other extraneous variables).^8,9^ Given these limitations, there remains a need to further investigate the effectiveness of PBRSS for mental health, substance use, and other general health outcomes.

### Defining peer support services

PBRSS are aimed at engaging individuals experiencing behavioral health conditions who need support to achieve and/or maintain stability in their community.^1^ This includes people with mental health conditions, substance use conditions, or some combination of both. PRS, who facilitate services, help reduce the severity of a condition, restore or improve an individual’s functioning, reduce reoccurrence of a condition, and promote long term recovery. The effectiveness of PRS is theorized to stem from the social support provided,^1,10,11^ modeling of positive social behaviors, upward social comparison, and fostering of optimism and experiential learning.^12^ The term “peer” is used to refer to all individuals with shared experience of addiction and recovery. Within PBRSS, those peers in stable recovery providing support may be referred to as a “peer leader,” “peer mentor,” “peer guide,” “peer recovery coach,” “peer resource specialist,” or “peer recovery specialist.” Peers may be volunteers or be employed following training and certification.^1,13^

Over the past decade, PBRSS have become an integral part of behavioral health care.^13^ Four major types of services are provided within PBRSS: (1) peer mentoring/coaching (e.g., a one-on-one relationship focused on motiving and supporting someone seeking recovery) in which PRS provide hope, validation, support, and assistance to accomplish goals; (2) connection to recovery resources (e.g., professional/nonprofessional in the community); (3) facilitating recovery groups (e.g., structured support groups, including sharing personal stories); and (4) building community (e.g., development of alternative social networks).^1^ Additionally, PRS support recovery planning to assist peers in setting their own goals, help them accomplish these goals, and help the peer function as a member of their own treatment/recovery team, facilitating their self-efficacy in the recovery process.^14^

### Impact of peer delivered support services

#### Substance use and harm reduction

Research investigating the effectiveness of PBRSS in reducing substance use has been promising across a variety of clinical settings.^5,8,9^ One systematic review found that, although findings are mixed, PRS have the potential to reduce substance use and relapse, increase treatment retention, and improve treatment satisfaction and relationships (e.g., with treatment providers and social supports).^8^ However, this review also noted the need to identify for whom and under what conditions PBRSS interventions are most useful and the potential cost-benefits to healthcare systems. Unfortunately, most studies include predominantly White, middle-aged, and male clients.^5,15,16^

A more recent study in Federally Qualified Health Centers found that persons who agreed to 9 months of PRS contact had significant reductions from intake to 6-month follow up in reported substance use including alcohol, cannabis, cocaine, heroin, benzodiazepines, and hallucinogens.^17^ Another study found PRS to be effective in engaging and connecting high risk individuals to resources post-discharge from the Emergency Department (ED), including referrals for outpatient or inpatient treatment, medication assisted programs, naloxone trainings, and other community-based services (e.g., food, transportation, housing).^18^ However, Webb et al.^16^ found that White patients, compared with Black patients, had a higher likelihood of being enrolled in SUD treatment by PRS after controlling for other important variables (e.g., age, sex, insurance). Similarly, Ashford et al.^3^ found that Latinx participants were less likely to have multiple engagements with peer-based supports. More research is needed accounting for the impact of demographics on outcomes.

#### Mental and physical health

A systematic review examining the impact of PBRSS across mental health, recovery, and physical health and wellness reported mixed findings.^19^ Treatment teams that included PRS were associated with psychosocial, satisfaction, clinical, or service use outcomes that were equivalent to those provided by non-peer providers employed in similar roles. Similarly, there was little to no evidence that peer services resulted in positive effects on hospitalization rates, overall symptom reduction, and service satisfaction. On the other hand, peer support positively impacted individuals’ self-management of their physical health (e.g., action planning, diet, and communication with physicians).^19^ Peer support also impacted participant health-related self-efficacy. In general, self-efficacy is theorized to motivate behavior engagement, with studies finding that self-efficacy can predict outcomes and/ or mediate treatment effects for substance use disorders.^20^ Finally, in another study, PBRSS were associated with increases in routine primary care visits and behavioral health access and utilization, reduced hospital inpatient days for physical concerns, fewer unplanned urgent outpatient medical visits at follow-up, and increases in clients’ satisfaction with their health.^17^

#### Other outcomes

Finally, research indicates that PRS instill hope, foster empowerment, improve quality of life,^19^ and reduce stigma associated with substance use and mental health difficulty.^21,22^ Additionally, a favorable relationship with a PRS can result in positive therapeutic outcomes including fewer psychiatric and health problems, and better family relationships.^23^

### Purpose of this study

Overall, evidence suggests that PBRSS have the potential of improving the lives of individuals who are looking to establish or maintain long-term recovery. PBRSS can lead to reductions in substance use, relapse, and hospitalizations rates, as well as reductions in psychiatric symptoms. The integration of PBRSS into various clinical settings (e.g., primary care, community settings, EDs) can lead to less costly services for the healthcare system and clients (e.g., less use of emergency services) and add to services provided by traditional providers. However, the current evidence base for PBRSS has significant methodological and statistical limitations (e.g., lack of rigorous statistical methods, control of extraneous variables). Few studies have focused on potential mediating factors or potential covariates that might also impact client outcomes.

Hence, the purpose of this study is to investigate the impact of PBRSS on client outcomes over time (i.e., two assessment time points) and the mediating factors that impact these outcomes, while controlling for important sociodemographic information. Specifically, while controlling for factors such as sex, race/ethnicity and age, this study aimed to examine the following: (1) the relationship between PBRSS and client outcomes (e.g., substance use, use of emergency services, use of outpatient services, overall health, and living situation) over two assessment points; and (2) whether this relationship is mediated by client self-efficacy or client perceptions of how PRS treat them (i.e., relationship with the PRS).

## Methods

### Data and design

Data were collected across 58 sites within 25 agencies certified to provide PBRSS that are delivered by PRS in a state located in Northeastern United States. These agencies were required to follow standard policies and procedures, quality improvement, and data collection and reporting standards as mandated by the Department of Behavioral Health. Agencies varied in the services they provide, with some being purely recovery-oriented (these are often non-profit, independent organizations headed by community leaders), and others offering standard therapy and crisis stabilization (often with a Chief Executive Officer, where the agency may be for-profit or not-for-profit). The use of de-identified data for this study was approved by the Institutional Review Board (IRB).

The state where the study took place certifies PRS that can be reimbursed through Medicaid. Certification involves a 46-h training focused on *advocacy* (e.g., to promote person-centered support services), *wellness and recovery* (e.g., assist the individual to identify and build on their strengths and resiliencies; assist individual in identifying basic needs), *mentoring and education* (e.g., serve as a role model for an individual; educate through shared experience), and *ethical responsibility* (e.g., recognize and maintain professional and personal boundaries; maintain current, accurate knowledge of trends and issues related to wellness and recovery). Certified PRS then complete a 500-h paid or unpaid internship to gain experience providing services. Following internship, PRS are eligible to take a 75-question multiple choice exam provided by the state’s Certification Board, to become certified. In addition, agencies have used other funding sources when available to pay for PRS, and therefore not all PRS are certified. This may arise, for example, when agencies determine that some PRS may be disadvantaged to get certified if they lack education or if English is a second language.

### Participants

Participants had to be 18 years old or older and be enrolled in services at one of the sites employing PRS. At baseline (i.e., time point 1 [T1]), *N* = 1544 completed the Recovery Oriented Measures Survey (ROMS; see *measures* below). Following baseline, *N* = 412 completed the ROMS at time point two (T2). Given interest in investigating the impact of peer services over two time periods, only participants who have data at both time points (*N* = 412) were included in the final sample. Of *N* = 412, most participants were White (70.6%), male (57.3%), and between the ages of 45 and 64 years (45.4%) (see Table 1 for more specific demographic information).

**Table 1:** Demographic information (*N* = 412)

	*n* (%)
Gender	
Male	236 (57.3)
Female	140 (34.0)
Transgender	2 (0.5)
Choose not to answer	11 (2.7)
Missing	23 (5.6)
Race/ethnicity	
Hispanic	20 (4.9)
White	291 (70.6)
Black	37 (9.0)
Asian	2 (0.5)
American Native/Alaskan Native	3 (0.7)
Multiracial	9 (2.2)
Other	12 (2.9)
Missing	38 (9.2)
Age	
18–24	11 (2.7)
25–44	159 (38.6)
45–64	187 (45.4)
65 and over	20 (4.9)
Choose not to answer	27 (6.6)
Missing	8 (1.9)

% = percent; *n* number

### Procedures

Individuals interested in or receiving services at one of the sites employing PRS were eligible if they wished to establish/maintain recovery and were also interested in being connected to a PRS. They were then scheduled for an intake to discuss needs and possible resources available. A second meeting was scheduled where the client completed the ROMS privately (see *measures* below). The length between the first and second meeting varied from days to weeks, depending on the client’s needs and desire to meet with the PRS. Clients generally completed this measure again 30 days after their second meeting with the PRS, followed by 60 days, and 90 days after that until the client was no longer receiving services.

### Measures

The Recovery Oriented Measures Survey (ROMS) is a 53-item questionnaire created by the funding agency and stakeholders across the state who were to implement the measure. This questionnaire assessed demographic information (e.g., race, age, income), services sought and received from PRS (e.g., “In which of the following areas are you seeking/have received support from a Peer Recovery Specialist?”), substance use in the past 30 days (e.g., “In the past 30 days, how many times have you used alcohol?”), and utilization of services (e.g., Hospital Emergency Department, urgent care) for mental health and substance use (e.g., “In the past 30 days, approximately how many times did you visit the Hospital Emergency Department for treatment for Alcohol or Substance Use?”). Furthermore, clients were asked their perceptions of how PRS treat them (e.g., “My Peer Recovery Specialist believes that I can grow, change, and recover”) and their self-efficacy in relation to recovery (e.g., “I feel that I can manage my health and recovery process”).

### Data preparation

#### Independent Variable (IV)

The IV of interest in this study was “Dosage” of PBRSS, or the areas in which PRS have been of help/provided support to clients. There were 24 response options (Cronbach’s α = 0.767; e.g., mental health services, substance use services, benefits applications, relapse prevention, etc.), with respondents answering yes (1), they received help/support in that area, or no (0), they did not receive help/support in that area. An overall score ranging from 0 to 24 was created per client measuring the number of overall services they received help/support in, with the IV measured as a continuous variable.

#### Mediators

There were two mediating factors of interest in this study: (1) client’s perception of PRS relationship/helpfulness (sample item: “My Peer Recovery Specialist helped me with decreasing self-stigma”), with nine items (Cronbach’s α = 0.939), rated on a Likert scale ranging from 1 (*strongly disagree*) to 5 (*strongly agree*). Higher scores indicate greater perceived helpfulness of and better relationship with their PRS. (2) Client’s self-efficacy (sample item: “I feel that I can manage my health and recovery process”), three items (Cronbach’s α = 0.857), rated on a Likert scale ranging from 1 (*strongly disagree*) to 5 (*strongly agree*). Higher scores on this scale are indicative of a higher sense of self-efficacy. For both mediators, an average was calculated ranging from 1 to 5.

#### Covariates

The covariates of interest in this study included: (1) race/ethnicity, measured as a categorical variable of 0 = White and 1 = Non-White or Hispanic, given the small sample size (see Table 1) for other racial groups and ethnicity (i.e., Hispanic, Black, Asian, Multiracial, American Native/ Alaskan Native, and other); (2) sex, measured as a categorical variable of 0 = male and 1 = female (individuals who identified as transgender were removed from analyses due to low sample size, *n* = 2); and (3) age, measured as a categorical variable of 0 = 18–44 years old and 1 = 45 and over.

#### Dependent Variables (DVs)

The outcomes of interest in this study were as follows: (1) client’s use of substances, consisting of 15 questions asking about substance use with the following stem: “In the past 30 days, how many times have you used…?” Substance categories include alcohol, cannabis, opiates, inhalants, hallucinogens, and an assortment of other substances. Alcohol use was assessed separately, with response options ranked so that 0 = none, 1 = 1, 2 = 2–4, 3 = 5–10, 4 = 11–15, 5 = 16–20, and 6 = more than 20, and an average score of alcohol use frequency created. All other substances (e.g., cannabis, opiates, hallucinogens) were grouped together into an unregulated substance use category and a score computed measuring the maximum frequency of unregulated substance use across these substances (e.g., if a participant reports using cannabis 16–20 times in the past 30 days and all other substance use frequency is lower than that [e.g., using cocaine 2–4 times, etc.], the maximum frequency was counted as 16–20 times, or a ranked score of 5).

(2) Next is client’s use of emergency services, consisting of four items assessing frequency of visits to the ER for either mental health or emotional issues, alcohol or substance use treatment, overdose, or other medical reasons (sample item: “In the past 30 days, approximately how many times did you visit the Hospital Emergency Department for treatment for Mental Health or Emotional Issues?”). Response options were ranked so that 0 = none, 1 = 1, 2 = 2–4, 3 = 5–10, 4 = 11–15, 5 = 16–20, and 6 = more than 20. After ranking, a score per client was computed to assess the maximum frequency of utilizing emergency services across all four items (e.g., if a client endorses the following ranked scores: ER for emotional issues = 1, ER for substance use treatment = 3, ER for an overdose = 2, and ER for a medical reason = 1, the frequency of using emergency services would be coded as a ranked score of 3, or 5–10 times). (3) Client’s use of outpatient services is made of items assessing frequency of receiving outpatient treatment for a physical illness or injury, mental health or emotional issues, alcohol or substance use treatment, or other medical treatment (sample item: “In the past 30 days, how many times did you receive outpatient treatment (non-emergency visits to a doctor or clinic or urgent care) for Physical Illness or Injury?”). Response options were ranked so that 0 = none, 1 = 1, 2 = 2–4, 3 = 5–10, 4 = 11–15, 5 = 16–20, and 6 = more than 20. Similar to the above, a score per client was computed to assess the maximum frequency of utilizing outpatient services across all four items. (4) Client’s overall health is one item rated on a Likert scale of 1 (*poor*) to 5 (*excellent*), measured as a continuous variable with higher scores suggesting a better overall health rating. (5) Client’s report of their living situation (e.g., homeless, independent living) was measured as a ranked variable of 0 = homelessness or shelter, 1 = residential treatment or recovery/sober house, and 2 = staying with a friend or independent living. Higher scores on this variable suggest a better living condition.

### Analytic plan

Preliminary analyses examined descriptive statistics, and data were analyzed for assumptions. While 1544 completed the ROMS, the sample in the current study is comprised of the *N* = 412 participants who completed the ROMS at T1 and T2. Therefore, independent samples *t*-tests compared baseline scores (substance use, outpatient/emergency services use, health ratings, living situation, self-efficacy, treatment by peers) between those in the current sample to those who were removed from the current study (*N* = 1132), and Chi-square tests were conducted at baseline for categorical variables (age, race/ethnicity, sex). For independent samples *t*-tests, Cohen’s *d* effect sizes were reported, with effect sizes considered small at 0.20, medium at 0.50, and large at 0.80.^24^ For chi-square tests, Phi coefficient (φ) effect sizes were reported, where φ = 0.1 is considered a small effect, 0.3 a medium effect, and 0.5 a large effect.^25^ *SPSS v.28* was used for generalizability analyses.^26^

Structural equation modeling (SEM)^27^ using cross-lagged panel models (CLPM)^28^ were used to test the longitudinal mediational models. Specifically, “half” longitudinal mediational CLPMs were conducted given the two waves of data. CLPMs allow for between differences to be assessed across time (e.g., individuals receiving a higher number of peer-based services show a larger reduction in alcohol use, compared to individuals with low number of services), compared with other models that require more waves of data and assess within-person differences (e.g., random intercepts CLPM).^29,30^

**Figure 1:**
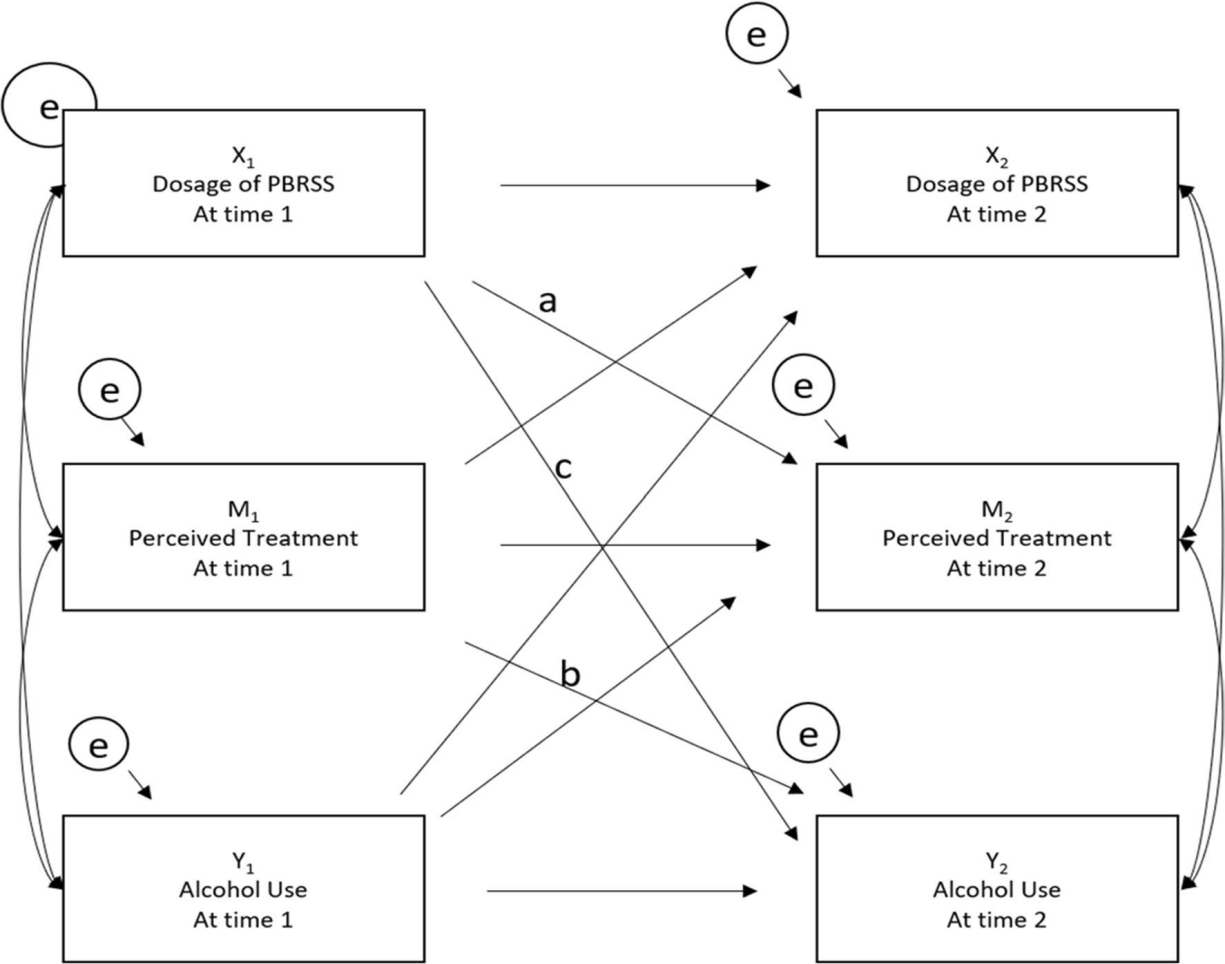
Example of cross-lagged panel longitudinal model tested. *Notes*. X_1_ = independent variable at T1; X_2_ = independent variable at T2; M_1_ = mediator at T1; M_2_ = mediator at T2; Y_1_ = dependent variable at T1; Y_2_ = dependent variable at T2; e = residuals. Covariates are not included in the figure to enhance figure clarity

See Fig. 1 for an example of one of the longitudinal mediational CLPM tested, with X representing dosage of PBRSS, M representing the mediator (e.g., PRS relationship/helpfulness), and Y representing the dependent variable (e.g., alcohol use). Although not included in the figure, the model also included covariates (e.g., age). Subscripts signify the time point in which these variables were measured; subscript one = T1 and subscript two = T2. Complex modeling was conducted using *RStudio* and the Lavaan package.^31^ Full Information Maximum Likelihood (FIML) was used as an estimation technique for missing data to estimate the model.^32^ Each variable was allowed to predict subsequent follow-up assessments of the same variable (e.g., dosage of PBRSS at T1 predicting dosage of PBRSS at T2) to assess autoregressive effects. All possible indirect pathways and effect sizes are reported, as well as standardized path coefficients for all paths. For mediation, steps to test this “half” longitudinal model followed Cole and Maxwell’s recommendations.^33^ First, a pair of longitudinal tests were conducted to (1) estimate Path a (see Fig. 1) in the regression of M_2_ onto X_1_, controlling for M_1_ and (2) estimate Path b in the regression of Y_2_ onto M_1_, controlling for Y_1_. The product of ab provided an estimate of the mediational effect of X on Y through M. To determine statistical significance of the proposed indirect effect of dosage of PBRSS on the outcome through the mediator, a 95% bias-corrected confidence interval from 5000 generated bootstrapped samples was tested.^33^ Model fit was evaluated with the comparative fit index (CFI), with higher scores between 0.0 and 1.0 indicating better fit, as well as root mean square error of approximation (RMSEA), with acceptable scores below 0.06.

Regarding statistical power, Kline^34^ recommends using the ratio of observations (participants) to estimated parameters (i.e., paths; *N*:q) as a guide in establishing statistical power for SEM. Specifically, Kline^34^ suggests that the N:q ratio should be 20:1, or 20 participants for each estimated path in the model. For this study, the largest model (with covariate[s]) tested included 17 paths, suggesting a sample size of 340, indicating that this study was well powered.

## Results

### Preliminary analyses

Means, standard deviations, skewness, and kurtosis for the IV, DVs, and mediators across T1 and T2 are presented in Table 2 to describe the sample. Overall, participants reported the following: receiving a higher number of peer-based services (i.e., dosage) at T2; better perceived relationship with/helpfulness of PRS at T2; a higher sense of self-efficacy at T2; a lower frequency of alcohol and unregulated substance use at T2; a lower frequency of receiving outpatient and emergency services at T2; and a higher/better overall health and living condition at T2.

**Table 2:** Descriptive statistics for study variables across time (*N* = 412)

Variable	T1				T2			
	*n*	M(SD)	Skew	Kurt	*n*	M(SD)	Skew	Kurt
Dosage of PBRSS	354	3.05 (2.69)	1.50	4.93	401	4.16 (2.65)	1.60	5.98
Perceived relationship/helpfulness	332	4.08 (0.69)	− 0.75	1.92	329	4.41 (0.58)	− 1.43	5.06
Self-efficacy	384	4.36 (0.65)	− 1.12	2.71	401	4.48 (0.60)	− 1.50	4.73
Alcohol use	403	1.35 (2.08)	1.32	0.29	403	0.84 (1.68)	2.07	3.25
Unregulated substance use	403	1.04 (1.87)	1.68	1.51	407	0.52 (1.48)	2.94	7.44
Emergency services	403	0.45 (0.64)	1.19	0.52	408	0.22 (0.45)	1.99	3.21
Outpatient services	400	0.80 (1.08)	0.98	− 0.54	407	0.57 (0.92)	1.42	0.77
Overall health	392	3.14 (0.96)	− 0.29	0.36	401	3.27 (0.94)	− 0.37	0.31
Living situation	331	1.68 (0.61)	− 1.77	1.91	348	1.77 (0.52)	− 2.24	4.13

*PBRSS* peer-based recovery support services. *n* number, *M* mean, *SD* standard deviation, *T1* time point 1, *T2* time point 2, *Skew* skewness, *Kurt* kurtosis

### Generalizability

Independent samples t-tests revealed several differences between participants not included in the sample (*N* = 1132) and participants included in the sample (*N* = 412). At initial assessment, participants not included in the sample received more peer-based services (M = 3.06, SD = 2.88) compared to participants who were included in the sample (M = 3.05, SD = 2.69), *t*(1321) = 0.07, and *p* = 0.04, although effect size was very small (*d* = 0.004). Participants not included in the sample reported a higher frequency of unregulated substance use (M = 1.50, SD = 2.23) compared with participants included in the sample (M = 1.04, SD = 1.87) *t*(1,515) = 3.70, and *p* < 0.001 (*d* = 0.22). Furthermore, participants who were included in the sample reported better living conditions (M = 1.69, SD = 0.61) compared with participants not included in the sample (M = 1.57, SD = 0.70), *t*(1,283) = − 2.64, *p* < 0.001 (*d* = − 0.17). Lastly, a chi-square test suggested differences in age for participants included and not included in the sample, *X*^2^ (1, *N* = 1255) = 7.93, and *p* = < 0.01 (φ = 0.08, small effect size). Of participants who were not included in the final sample size, about 54% were 18–44 years old and about 46% were 45 years old or older. In contrast, of participants who were included in the final sample, about 45% were 18–44 years old, and about 55% were 45 years old or older. No significant differences were found between samples regarding sex, race/ethnicity, PRS relationship/helpfulness, self-efficacy, alcohol use, overall health, emergency services use, and outpatient services use.

### Complex modeling

Given the 6 outcomes of interest in this study, 2 mediators, and one independent variable, 12 models were run with a Bonferroni^35^ adjusted alpha level of 0.00417 per model (0.05/12). See Table 3 for unstandardized and standardized path coefficients, *p* values, and lower and upper 95% confidence intervals (CI) for all paths. Model fit information can also be found in Table 3. A summary of significant results based on the adjusted alpha level is provided here. However, both significant and non-significant results based on the adjusted alpha level are reported in Table 3.

**Table 3:** Results from cross-lagged panel models

	B	β	*p*	95% CI lower	95% CI upper
Model 1 (DV, alcohol use; M, relationship/helpfulness)*					
*Autoregressive Paths*					
T1 Dosage of PBRRS → T2 Dosage of PBRRS	0.57	0.58	.000	0.44	0.69
T1 Relationship/Helpfulness → T2 Relationship/Helpfulness	0.23	0.27	.000	0.20	0.38
T1 Alcohol Use → T2 Alcohol Use	0.27	0.34	.000	0.17	0.38
*Cross-Lagged Effects*					
T1 Dosage of PBRRS → T2 Relationship/Helpfulness (Path a)	0.04	0.20	.001	0.01	0.08
T1 Relationship/Helpfulness → T2 Alcohol Use (Path b)	− 0.05	− 0.02	.757	− 0.37	0.26
T1 Dosage of PBRRS → T2 Alcohol Use (Path c)	− 0.01	− 0.01	.845	− 0.07	0.06
T1 Relationship/Helpfulness → T2 Dosage of PBRRS	− 0.79	− 0.21	.001	− 1.43	− 0.23
T1 Alcohol Use → T2 Dosage of PBRRS	0.00	0.00	.989	− 0.11	0.10
T1 Alcohol Use → T2 Relationship/Helpfulness	0.00	0.05	.350	− 0.01	0.04
a*b	− 0.00	− 0.00	.757	− 0.02	0.01
*Intercept Associations*					
T1 Dosage of PBRSS ↔ T1 Relationship/Helpfulness	0.84	0.46	.000	0.65	1.06
T1 Dosage of PBRSS ↔ T1 Alcohol Use	− 0.50	− 0.09	.090	− 1.05	0.06
T1 Relationship/Helpfulness ↔ T1 Alcohol Use	− 0.23	− 0.16	.003	− 0.40	− 0.07
T2 Dosage of PBRSS ↔ T2 Relationship/Helpfulness	0.35	0.29	.000	0.19	0.53
T2 Dosage of PBRSS ↔ T2 Alcohol Use	− 0.14	− 0.04	.453	− 0.45	0.17
T2 Relationship/Helpfulness ↔ T2 Alcohol Use	− 0.07	− 0.09	.148	− 0.17	0.03
*Model Fit Indices*	CFI = 1.00; TLI = 1.00; RMSEA = 0.000 (0.000,0.000^a^); SRMR = 0.000				
Model 2 (DV: Alcohol Use, M: Self-Efficacy)*					
*Autoregressive Paths*					
T1 Dosage of PBRSS → T2 Dosage of PBRSS	0.49	0.49	.000	0.36	0.60
T1 Self-Efficacy → T2 Self-Efficacy	0.31	0.33	.000	0.20	0.44
T1 Alcohol Use → T2 Alcohol Use	0.27	0.33	.000	0.17	0.37
*Cross-Lagged Effects*					
T1 Dosage of PBRSS → T2 Self-Efficacy (Path a)	0.03	0.13	.006	0.01	0.06
T1 Self-Efficacy → T2 Alcohol Use (Path b)	− 0.18	− 0.07	.150	− 0.51	0.12
T1 Dosage of PBRSS → T2 Alcohol Use (Path c)	− 0.00	− 0.01	.897	− 0.06	0.06
T1 Self-Efficacy → T2 Dosage of PBRSS	− 0.10	− 0.03	.633	− 0.58	0.33
T1 Alcohol Use → T2 Dosage of PBRSS	0.02	0.02	.661	− 0.08	0.13
T1 Alcohol Use → T2 Self-Efficacy	0.01	0..02	.670	− 0.02	0.03
a*b	− 0.01	− 0.01	.202	− 0.02	0.00
*Intercept Associations*					
T1 Dosage of PBRSS ↔ T1 Self-Efficacy	0.10	0.06	.294	− 0.08	0.29
T1 Dosage of PBRSS ↔ T1 Alcohol Use	− 0.49	− 0.09	.103	− 1.03	0.06
T1 Self-Efficacy ↔ T1 Alcohol Use	− 0.25	− 0.19	.000	− 0.42	0.10
T2 Dosage of PBRSS ↔ T2 Self-Efficacy	0.27	0.21	.000	0.13	0.42
T2 Dosage of PBRSS ↔ T2 Alcohol Use	− 0.16	− 0.05	.383	− 0.48	0.15
T2 Self-Efficacy ↔ T2 Alcohol Use	− 0.17	− 0.19	.000	− 0.27	− 0.06
*Model Fit Indices*	CFI = 1.00; TLI = 1.00; RMSEA = 0.000 (0.000,0.000^a^); SRMR = 0.00				
Model 3 (DV: Unregulated SU, M: Relationship/Helpfulness)*					
*Autoregressive Paths*					
T1 Dosage of PBRSS → T2 Dosage of PBRSS	0.57	0.58	.000	0.44	0.69
T1 Relationship/Helpfulness → T2 Relationship/Helpfulness	0.23	0.27	.000	0.07	0.40
T1 Unregulated SU → T2 Unregulated SU	0.29	0.36	.000	0.19	0.40
*Cross-Lagged Effects*					
T1 Dosage of PBRSS → T2 Relationship/Helpfulness (Path a)	0.04	0.20	.001	0.01	0.08
T1 Relationship/Helpfulness → T2 Unregulated SU (Path b)	0.08	0.04	.546	− 0.20	0.37
T1 Dosage of PBRSS → T2 Unregulated SU (Path c)	0.05	0.09	.126	− 0.05	0.14
T1 Relationship/Helpfulness → T2 Dosage of PBRSS	− 0.82	− 0.22	.000	− 1.47	− 0.27
T1 Unregulated SU → T2 Dosage of PBRSS	− 0.03	− 0.02	.601	− 0.15	0.09
T1 Unregulated SU → T2 Relationship/Helpfulness	− 0.00	− 0.01	.821	− 0.04	0.03
a*b	0.00	0.01	.557	− 0.01	0.02
*Intercept Associations*					
T1 Dosage of PBRSS ↔ T1 Relationship/Helpfulness	0.83	0.45	.000	0.64	1.05
T1 Dosage of PBRSS ↔ T1 Unregulated SU	− 0.08	− 0.02	.757	− 0.61	0.46
T1 Relationship/Helpfulness ↔ T1 Unregulated SU	− 0.16	− 0.13	.020	− 0.32	− 0.01
T2 Dosage of PBRSS ↔ T2 Relationship/Helpfulness	0.36	0.30	.000	0.19	0.54
T2 Dosage of PBRSS ↔ T2 Unregulated SU	0.18	0.06	.275	− 0.14	0.48
T2 Relationship/Helpfulness ↔ T2 Unregulated SU	− 0.13	− 0.13	.003	− 0.27	− 0.01
*Model Fit Indices*	CFI = 1.00; TLI = 1.00; RMSEA = 0.000 (0.000, 0.000^a^); SRMR = 0.00				
Model 4 (DV: Unregulated SU, M: Self-Efficacy)*					
*Autoregressive Paths*					
T1 Dosage of PBRSS → T2 Dosage of PBRSS	0.49	0.49	.000	0.36	0.60
T1 Self-Efficacy → T2 Self-Efficacy	0.31	0.33	.000	0.20	0.45
T1 Unregulated SU → T2 Unregulated SU	0.28	0.36	.000	0.18	0.40
*Cross-Lagged Effects*					
T1 Dosage of PBRSS → T2 Self-Efficacy (Path a)	0.03	0.13	.007	0.01	0.06
T1 Self-Efficacy → T2 Unregulated SU (Path b)	− 0.08	− 0.03	.487	− 0.34	0.17
T1 Dosage of PBRSS → T2 Unregulated SU (Path c)	0.06	0.10	.030	− 0.02	0.13
T1 Self-Efficacy→ T2 Dosage of PBRSS	− 0.14	− 0.04	.484	− 0.62	0.28
T1 Unregulated SU → T2 Dosage of PBRSS	− 0.01	− 0.00	.929	− 0.12	0.12
T1 Unregulated SU → T2 Self-Efficacy	0.00	0.01	.786	− 0.03	0.03
a*b	− 0.00	− 0.00	.499	− 0.01	0.01
*Intercept Associations*					
T1 Dosage of PBRSS ↔ T1 Self-Efficacy	0.12	0.06	.274	− 0.07	0.29
T1 Dosage of PBRSS ↔ T1 Unregulated SU	− 0.06	− 0.01	.832	− 0.57	0.48
T1 Self-Efficacy ↔ T1 Unregulated SU	− 0.13	− 0.11	.035	− 0.28	0.01
T2 Dosage of PBRSS ↔ T2 Self-Efficacy	0.27	0.21	.000	0.13	0.42
T2 Dosage of PBRSS ↔ T2 Unregulated SU	0.14	0.04	.390	− 0.18	0.46
T2 Self-Efficacy ↔ T2 Unregulated SU	− 0.15	− 0.20	.000	− 0.29	− 0.04
*Model Fit Indices*	CFI = 1.00; TLI = 1.00; RMSEA = 0.000 (0.000, 0.000^a^); SRMR = 0.00				
Model 5 (DV: Emergency Services, M: Relationship/Helpfulness)*					
*Autoregressive Paths*					
T1 Dosage of PBRSS → T2 Dosage of PBRSS	0.58	0.58	.000	0.44	0.70
T1 Relationship/Helpfulness → T2 Relationship/Helpfulness	0.23	0.27	.000	0.07	0.40
T1 Emergency Services → T2 Emergency Services	0.13	0.18	.000	0.05	0.22
*Cross-Lagged Effects*					
T1 Dosage of PBRSS → T2 Relationship/Helpfulness (Path a)	0.04	0.20	.001	0.01	0.08
T1 Relationship/Helpfulness → T2 Emergency Services (Path b)	− 0.06	− 0.08	.171	− 0.14	0.03
T1 Dosage of PBRSS → T2 Emergency Services (Path c)	0.03	0.16	.006	0.01	0.05
T1 Relationship/Helpfulness → T2 Dosage of PBRSS	− 0.81	− 0.21	.001	− 1.44	− 0.26
T1 Emergency Services → T2 Dosage of PBRSS	0.23	0.06	.204	− 0.13	0.60
T1 Emergency Services → T2 Relationship/Helpfulness	0.04	0.04	.451	− 0.05	0.13
a*b	− 0.00	− 0.02	.207	− 0.01	0.00
*Intercept Associations*					
T1 Dosage of PBRSS ↔ T1 Relationship/Helpfulness	0.84	0.45	.000	0.64	1.06
T1 Dosage of PBRSS ↔ T1 Emergency Services	0.01	0.01	.877	− 0.17	0.20
T1 Relationship/Helpfulness ↔ T1 Emergency Services	− 0.01	− 0.02	.764	− 0.05	0.04
T2 Dosage of PBRSS ↔ T2 Relationship/Helpfulness	0.35	0.29	.000	0.18	0.53
T2 Dosage of PBRSS ↔ T2 Emergency Services	− 0.02	− 0.02	.769	− 0.11	0.08
T2 Relationship/Helpfulness ↔ T2 Emergency Services	0.00	0.00	.989	− 0.02	0.02
*Model Fit Indices*	CFI = 1.00; TLI = 1.00; RMSEA = 0.000 (0.000, 0.000^a^); SRMR = 0.00				
Model 6 (DV: Emergency Services, M: Self-Efficacy)*					
*Autoregressive Paths*					
T1 Dosage of PBRSS → T2 Dosage of PBRSS	0.49	0.49	.000	0.36	0.60
T1 Self-Efficacy → T2 Self-Efficacy	0.31	0.33	.000	0.19	0.45
T1 Emergency Services → T2 Emergency Services	0.13	0.18	.000	0.05	0.22
*Cross-Lagged Effects*					
T1 Dosage of PBRSS → T2 Self-Efficacy (Path a)	0.03	0.14	.006	0.01	0.06
T1 Self-Efficacy → T2 Emergency Services (Path b)	− 0.04	− 0.06	.242	− 0.12	0.03
T1 Dosage of PBRSS → T2 Emergency Services (Path c)	0.02	0.13	.011	0.00	0.04
T1 Self-Efficacy → T2 Dosage of PBRSS	− 0.10	− 0.02	.636	− 0.58	0.33
T1 Emergency Services → T2 Dosage of PBRSS	0.27	0.07	.146	− 0.11	0.66
T1 Emergency Services → T2 Self-Efficacy	0.02	0.02	.740	− 0.08	0.11
a*b	− 0.00	− 0.01	.278	− 0.01	0.00
*Intercept Associations*					
T1 Dosage of PBRSS ↔ T1 Self-Efficacy	0.11	0.06	.261	− 0.07	0.30
T1 Dosage of PBRSS ↔ T1 Emergency Services	− 0.01	− 0.01	.892	− 0.20	0.18
T1 Self-Efficacy ↔ T1 Emergency Services	− 0.04	− 0.10	.051	− 0.08	0.00
T2 Dosage of PBRSS ↔ T2 Self-Efficacy	0.27	0.21	.000	0.13	0.42
T2 Dosage of PBRSS ↔ T2 Emergency Services	− 0.01	− 0.01	.920	− 0.10	0.09
T2 Self-Efficacy ↔ T2 Emergency Services	− 0.02	− 0.07	.157	− 0.04	0.00
*Model Fit Indices*	CFI = 1.00; TLI = 1.00; RMSEA = 0.000 (0.000, 0.000^a^); SRMR = 0.00				
Model 7 (DV: Outpatient Services, M: Relationship/Helpfulness)					
*Autoregressive Paths*					
T1 Dosage of PBRSS → T2 Dosage of PBRSS	0.58	0.59	.000	0.448	0.701
T1 Relationship/Helpfulness → T2 Relationship/Helpfulness	0.22	0.27	.000	0.073	0.389
T1 Outpatient Services → T2 Outpatient Services	0.10	0.12	.014	0.014	0.197
*Cross-Lagged Effects*					
T1 Dosage of PBRSS → T2 Relationship/Helpfulness (Path a)	0.05	0.22	.000	0.019	0.083
T1 Relationship/Helpfulness → T2 Outpatient Services (Path b)	− 0.17	− 0.13	.029	− 0.333	− 0.013
T1 Dosage of PBRSS → T2 Outpatient Services (Path c)	0.03	0.09	.148	− 0.017	0.072
T1 Relationship/Helpfulness → T2 Dosage of PBRSS	− 0.85	− 0.22	.000	− 1.513	− 0.310
T1 Outpatient Services → T2 Dosage of PBRSS	− 0.03	− 0.01	.813	− 0.236	0.185
T1 Outpatient Services → T2 Relationship/Helpfulness	− 0.04	− 0.07	.219	− 0.098	0.027
Gender → T2 Relationship/Helpfulness	0.12	0.10	.048	− 0.004	0.239
Gender → T2 Outpatient Services	0.20	0.11	.038	0.005	0.402
a*b	− 0.01	− 0.03	.067	− 0.020	0.000
*Intercept Associations*					
T1 Dosage of PBRSS ↔ T1 Relationship/Helpfulness	0.84	0.45	.000	0.646	1.056
T1 Dosage of PBRSS ↔ T1 Outpatient Services	0.37	0.13	.015	0.036	0.706
T1 Relationship/Helpfulness ↔ T1 Outpatient Services	0.01	0.01	.881	− 0.071	0.080
T2 Dosage of PBRSS ↔ T2 Relationship/Helpfulness	0.35	0.30	.000	0.186	0.530
T2 Dosage of PBRSS ↔ T2 Outpatient Services	0.19	0.10	.072	− 0.053	0.464
T2 Relationship/Helpfulness ↔ T2 Outpatient Services	0.04	0.09	.151	− 0.008	0.082
*Model Fit Indices*	CFI = 0.972; TLI = 0.854; RMSEA = 0.068 (0.025, 0.116^a^); SRMR = 0.036				
Model 8 (DV: Outpatient Services, M: Self-Efficacy)					
*Autoregressive Paths*					
T1 Dosage of PBRSS → T2 Dosage of PBRSS	0.49	0.49	.000	0.36	0.60
T1 Self-Efficacy → T2 Self-Efficacy	0.31	0.33	.000	0.20	0.44
T1 Outpatient Services → T2 Outpatient Services	0.11	0.13	.008	0.02	0.20
*Cross-Lagged Effects*					
T1 Dosage of PBRSS → T2 Self-Efficacy (Path a)	0.03	0.14	.004	0.01	0.06
T1 Self-Efficacy → T2 Outpatient Services (Path b)	− 0.07	− 0.05	.351	− 0.22	0.09
T1 Dosage of PBRSS → T2 Outpatient Services (Path c)	0.01	0.03	.549	− 0.03	0.05
T1 Self-Efficacy → T2 Dosage of PBRSS	− 0.16	− 0.04	.422	− 0.66	0.26
T1 Outpatient Services → T2 Dosage of PBRSS	0.01	0.00	.937	− 0.20	0.22
T1 Outpatient Services → T2 Self-Efficacy	0.00	0.00	.993	− 0.06	0.06
Gender → T2 Self-Efficacy	0.09	0.08	.112	− 0.03	0.21
Gender → T2 Outpatient Services	0.21	0.11	.037	0.00	0.41
a*b	− 0.00	− 0.01	.375	− 0.01	0.00
*Intercept Associations*					
T1 Dosage of PBRSS ↔ T1 Self-Efficacy	0.11	0.06	.261	− 0.07	0.30
T1 Dosage of PBRSS ↔ T1 Outpatient Services	0.37	0.13	.017	0.03	0.70
T1 Self-Efficacy ↔ T1 Outpatient Services	0.03	0.05	.376	− 0.04	0.10
T2 Dosage of PBRSS ↔ T2 Self-Efficacy	0.27	0.21	.000	0.13	0.42
T2 Dosage of PBRSS ↔ T2 Outpatient Services	0.24	0.12	.027	− 0.02	0.53
T2 Self-Efficacy ↔ T2 Outpatient Services	0.02	0.05	.355	− 0.02	0.07
*Model Fit Indices*	CFI = 0.962; TLI = 0.798; RMSEA = 0.066 (0.022, 0.114^a^); SRMR = 0.031				
Model 9 (DV: Overall Health, M: Relationship/Helpfulness)					
*Autoregressive Paths*					
T1 Dosage of PBRSS → T2 Dosage of PBRSS	0.58	0.58	.000	0.45	0.70
T1 Relationship/Helpfulness → T2 Relationship/Helpfulness	0.19	0.23	.001	0.03	0.37
T1 Overall Health → T2 Overall Health	0.31	0.33	.000	0.21	0.42
*Cross-Lagged Effects*					
T1 Dosage of PBRSS → T2 Relationship/Helpfulness (Path a)	0.05	0.21	.001	0.02	0.08
T1 Relationship/Helpfulness → T2 Overall Health (Path b)	0.04	0.03	.659	− 0.13	0.21
T1 Dosage of PBRSS → T2 Overall Health (Path c)	− 0.00	− 0.01	.847	− 0.06	0.05
T1 Relationship/Helpfulness → T2 Dosage of PBRSS	− 0.91	− 0.24	.000	− 1.57	− 0.35
T1 Overall Health → T2 Dosage of PBRSS	0.12	0.04	.352	− 0.11	0.35
T1 Overall Health → T2 Relationship/Helpfulness	0.04	0.07	.210	− 0.03	0.11
Age → T2 Relationship/Helpfulness	0.07	0.06	.228	− 0.06	0.21
Age → T2 Overall Health	− 0.40	− 0.21	.000	− 0.58	− 0.21
a*b	0.00	0.01	.662	− 0.01	0.01
*Intercept Associations*					
T1 Dosage of PBRSS ↔ T1 Relationship/Helpfulness	0.84	0.45	.000	0.64	1.06
T1 Dosage of PBRSS ↔ T1 Overall Health	0.35	0.14	.010	0.08	0.63
T1 Relationship/Helpfulness ↔ T1 Overall Health	0.16	0.24	.000	0.09	0.24
T2 Dosage of PBRSS ↔ T2 Relationship/Helpfulness	0.36	0.30	.000	0.19	0.54
T2 Dosage of PBRSS ↔ T2 Overall Health	0.10	0.05	.308	− 0.13	0.33
T2 Relationship/Helpfulness ↔ T2 Overall Health	0.04	0.08	.167	− 0.02	0.09
*Model Fit Indices*	CFI = 0.944; TLI = 0.704; RMSEA = 0.110 (0.070, 0.155^a^); SRMR = 0.044				
Model 10 (Overall Health, M: Self-Efficacy)					
*Autoregressive Paths*					
T1 Dosage of PBRSS → T2 Dosage of PBRSS	0.49	0.49	.000	0.36	0.60
T1 Self-Efficacy → T2 Self-Efficacy	0.30	0.32	.000	0.18	0.44
T1 Overall Health → T2 Overall Health	0.31	0.32	.000	0.21	0.41
*Cross-Lagged Effects*					
T1 Dosage of PBRSS → T2 Self-Efficacy (Path a)	0.03	0.13	.009	0.00	0.06
T1 Self-Efficacy → T2 Overall Health (Path b)	0.05	− 0.04	.473	− 0.09	0.19
T1 Dosage of PBRSS → T2 Overall Health (Path c)	0.00	0.00	.995	− 0.05	0.05
T1 Self-Efficacy → T2 Dosage of PBRSS	− 0.13	− 0.03	.527	− 0.62	0.31
T1 Overall Health → T2 Dosage of PBRSS	0.04	0.02	.751	− 0.20	0.27
T1 Overall Health → T2 Self-Efficacy	0.03	0.05	.331	− 0.03	0.10
Age → T2 Self-Efficacy	− 0.02	− 0.02	.741	− 0.14	0.10
Age → T2 Overall Health	− 0.40	− 0.22	.000	− 0.59	− 0.21
a*b	0.00	0.01	.487	− 0.00	0.01
*Intercept Associations*					
T1 Dosage of PBRSS ↔ T1 Self-Efficacy	0.12	0.06	.264	− 0.07	0.30
T1 Dosage of PBRSS ↔ T1 Overall Health	0.29	0.12	.032	0.02	0.57
T1 Self-Efficacy ↔ T1 Overall Health	0.13	0.21	.000	0.06	0.21
T2 Dosage of PBRSS ↔ T2 Self-Efficacy	0.26	0.21	.000	0.13	0.42
T2 Dosage of PBRSS ↔ T2 Overall Health	0.09	0.05	.370	− 0.15	0.32
T2 Self-Efficacy ↔ T2 Overall Health	0.12	0.26	.000	0.07	0.18
*Model Fit Indices*	CFI = 0.930; TLI = 0.635; RMSEA = 0.112 (0.072, 0.155^a^); SRMR = 0.044				
Model 11 (DV: Living Situation, M: Relationship/Helpfulness)					
*Autoregressive Paths*					
T1 Dosage of PBRSS → T2 Dosage of PBRSS	0.58	0.58	.000	0.45	0.69
T1 Relationship/Helpfulness → T2 Relationship/Helpfulness	0.23	0.26	.000	0.08	0.39
T1 Living Situation → T2 Living Situation	0.51	0.60	.000	0.36	0.65
*Cross-Lagged Effects*					
T1 Dosage of PBRSS → T2 Relationship/Helpfulness (Path a)	0.05	0.21	.000	0.02	0.08
T1 Relationship/Helpfulness → T2 Living Situation (Path b)	− 0.06	− 0.08	.178	− 0.15	0.03
T1 Dosage of PBRSS → T2 Living Situation (Path c)	− 0.01	− 0.03	.528	− 0.03	0.03
T1 Relationship/Helpfulness → T2 Dosage of PBRSS	− 0.81	− 0.21	.001	− 1.42	0.28
T1 Living Situation → T2 Dosage of PBRSS	− 0.13	− 0.03	.556	− 0.68	0.37
T1 Living Situation → T2 Relationship/Helpfulness	0.08	0.08	.137	− 0.02	0.18
Gender → T2 Relationship/Helpfulness	0.14	0.12	.023	0.02	0.26
Gender → T2 Living Situation	− 0.04	− 0.04	.418	− 0.15	0.07
a*b	− 0.00	− 0.02	.221	− 0.01	0.00
*Intercept Associations*					
T1 Dosage of PBRSS ↔ T1 Relationship/Helpfulness	0.84	0.45	.000	0.65	1.06
T1 Dosage of PBRSS ↔ T1 Living Situation	0.02	0.01	.822	− 0.23	0.26
T1 Relationship/Helpfulness ↔ T1 Living Situation	0.04	0.10	.085	− 0.01	0.09
T2 Dosage of PBRSS ↔ T2 Relationship/Helpfulness	0.37	0.31	.000	0.20	0.55
T2 Dosage of PBRSS ↔ T2 Living Situation	− 0.13	− 0.14	.028	− 0.33	0.04
T2 Relationship/Helpfulness ↔ T2 Living Situation	0.05	0.22	.001	0.01	0.09
*Model Fit Indices*	CFI = 0.986; TLI = 0.925; RMSEA = 0.058 (0.005, 0.107^a^); SRMR = 0.037				
Model 12 (DV: Living Situation, M: Self-Efficacy)*					
*Autoregressive Paths*					
T1 Dosage of PBRSS → T2 Dosage of PBRSS	0.49	0.49	.000	0.37	0.60
T1 Self-Efficacy → T2 Self-Efficacy	0.30	0.33	.000	0.19	0.44
T1 Living Situation → T2 Living Situation	0.50	0.59	.000	0.35	0.65
*Cross-Lagged Effects*					
T1 Dosage of PBRSS → T2 Self-Efficacy (Path a)	0.03	0.14	.006	0.01	0.06
T1 Self-Efficacy → T2 Living Situation (Path b)	− 0.03	− 0.04	.397	− 0.10	0.04
T1 Dosage of PBRSS → T2 Living Situation (Path c)	− 0.01	− 0.05	.309	− 0.04	0.02
T1 Self-Efficacy → T2 Dosage of PBRSS	− 0.15	− 0.04	.459	− 0.62	0.27
T1 Living Situation → T2 Dosage of PBRSS	− 0.21	− 0.05	.335	− 0.77	0.29
T1 Living Situation → T2 Self-Efficacy	0.08	0.09	.090	− 0.01	0.19
a*b	− 0.00	− 0.01	.421	− 0.00	0.00
*Intercepts Associations*					
T1 Dosage of PBRSS ↔ T1 Self-Efficacy	0.10	0.06	.284	− 0.08	0.29
T1 Dosage of PBRSS ↔ T1 Living Situation	0.00	0.00	.990	− 0.26	0.25
T1 Self-Efficacy ↔ T1 Living Situation	− 0.01	− 0.04	.535	− 0.06	0.03
T2 Dosage of PBRSS ↔ T2 Self-Efficacy	0.27	0.21	.000	0.14	0.42
T2 Dosage of PBRSS ↔ T2 Living Situation	− 0.11	− 0.12	.059	− 0.32	0.06
T2 Self-Efficacy ↔ T2 Living Situation	0.04	0.16	.010	0.00	0.07
*Model Fit Indices*	CFI = 1.00; TLI = 1.00; RMSEA = 0.000 (0.000, 0.000^a^); SRMR = 0.00				

*Covariates were removed from model because they were not statistically significant. Relationships are considered statistically significant at a Bonferroni adjusted alpha level of .00417. ^a^RMSEA reports 90% confidence interval. *DV* dependent variable, *M* mediator, *T1* time point 1, *T2* time point 2, *PBRSS* Peer-Based Recovery Support Services, *B* unstandardized beta weight, *β* standardized beta weight, *p P* value, *CI* confidence interval, *CFI* comparative fit index, *TLI* Tucker-Lewis Index, *RMSEA* root mean square error approximation, *SRMR* standardized root mean square residual

Results from autoregressive pathways indicated that most variables were related at T1 and T2. For example, a higher T1 dosage of PBRSS significantly predicted a higher T2 dosage of PBRSS (*p* < 0.001), a better T1 PRS relationship/helpfulness significantly predicted better T2 PRS relationship/helpfulness (*p* = 0.001), and a higher T1 self-efficacy significantly predicted a higher T2 self-efficacy (*p* < 0.001). Similar findings were assessed with outcomes (e.g., a higher frequency of T1 alcohol use significantly predicted a higher frequency of T2 alcohol use [models 1–2; *p* < 0.001]). However, after adjusting for alpha, T1 outpatient services did not significantly predict T2 outpatient services (models 7–8; *p* = 0.014 and 0.008, respectively).

Examining cross-lagged effects, results suggested that a higher T1 dosage of PBRSS significantly predicted a better perceived relationship with/helpfulness of PRS at T2 (models 1, 3, 5, 7, 9, and 11, path a; *p* = 0.001). Across all models that examined PRS relationship/helpfulness as the mediator (models 1, 3, 5, 7, 9, 11), a better T1 PRS relationship/helpfulness significantly predicted lower T2 dosage of PBRSS (*p* values ranging from < 0.001 to 0.001). Furthermore, older age significantly predicted a worse T2 overall health (models 9 & 10; *p* < 0.001). The cross lagged panel model examining frequency of outpatient services use as the DV and PRS helpfulness/relationship as the mediator (model 7) also revealed that a higher T1 dosage of PBRSS significantly predicted better T2 PRS relationship/helpfulness (path a, *p* < 0.001).

Finally, several covariate associations were found across all models where relationship with/helpfulness of PRS was treated as the mediator (models 1, 3, 5, 7, 9, 11). Specifically, a higher T1 dosage of PBRSS was significantly associated with better T1 PRS relationship/helpfulness (*p* < 0.001), and higher T2 dosage of PBRSS was significantly associated with better T2 PRS relationship/helpfulness (*p* < 0.001). Several statistically significant associations were also found between PRS relationship/helpfulness as the mediator and outcomes examined. Specifically, better T1 PRS relationship/helpfulness was significantly associated with a lower frequency of T1 alcohol use (model 1; *p* = 0.003) and a higher T1 overall health (model 9; *p* < 0.001). Lastly, better T2 PRS relationship/helpfulness was significantly associated with a lower frequency of T2 unregulated substance use (model 3; *p* = 0.003) and a better T2 living condition (model 11; *p* = 0.001).

Across all models where self-efficacy was treated as the mediator (models 2, 4, 6, 8, 10, 12), higher T2 dosage of PBRSS was significantly related to higher T2 self-efficacy (*p* < 0.001). Also, results showed that higher T1 self-efficacy was significantly associated with lower frequency of T1 alcohol use (model 2; *p* < 0.001) and higher T1 overall health (model 10; *p* < 0.001). A higher T2 self-efficacy was significantly associated with a lower frequency of T2 alcohol use (model 2; *p* < 0.001), a lower frequency of T2 unregulated substance use (model 4, *p* < 0.001), and a higher T2 overall health rating (model 10; *p* < 0.001).

## Discussion

This study examined the impact of PBRSS on client outcomes (alcohol and unregulated substance use, emergency and outpatient services use, overall health and living conditions), and whether client self-efficacy or perceived helpfulness of and relationship with a PRS mediated the associations between PBRSS and client outcomes. Outcomes in this study were analyzed over two time periods, and although several differences were found between clients who were included in the final sample compared with clients who were not (in terms of service dosage, unregulated substance use, living conditions, age), effect sizes for these differences were small (Cohen’s *d* ranged between 0.004 and 0.22; φ = 0.08). This suggests no practically meaningful difference between these samples, supporting the generalizability of results in this study.

Some findings in this study were expected, including autoregressive effects found in Table 3. For example, after applying a conservative alpha correction, constructs generally were significantly related across time (e.g., PRS dosage at T1 was significantly related to dosage at T2). Similarly, many point-in-time associations for client self-efficacy were expected (see Table 3, intercept associations), including the positive associations between self-efficacy and overall health at both initial and follow-up assessments, the negative associations between self-efficacy and alcohol use (i.e., less alcohol use) at both time-points, and the negative association between self-efficacy and unregulated substance use (i.e., less unregulated substance use) at follow-up. Self-efficacy has been shown to be associated with multiple health outcomes,^15,36,37^ supporting current associations found in this study. At follow-up assessment, peer services dose and self-efficacy were positively associated, suggesting that receiving more services from PRS fosters a greater sense of self-efficacy in clients. One study found that participants who were moderate to high attenders of peer-based support services showed statistically significant improvements over time in self-esteem/self-efficacy.^37^

Similar to findings regarding self-efficacy, better PRS relationship/helpfulness at initial assessment was associated with less alcohol use and better health, whereas at follow-up, PRS relationship/helpfulness was positively associated with a better living situation and negatively associated with unregulated substance use. This is consistent with literature suggesting that PRS impact clients by cultivating a relationship with them and providing social support.^23^ After applying a strict alpha correction, peer services dose was positively associated with better relationship/helpfulness ratings of the PRS at both initial and follow-up assessments (Table 3, intercept associations). Importantly, initially receiving more peer-based services significantly predicted better PRS relationship/helpfulness at a later time. Peer services included mental health/substance use treatment, community re-entry, employment/housing services, basic needs, and more. As compared to clients who do not receive a diversity of services, clients who receive such assistance may perceive the PRS as being more helpful and attending more to their relationship. This is consistent with research in which peer-based services improved relationships with both treatment providers and social supports.^9^

Client perceptions of initially having a good relationship with a helpful PRS significantly predicted less use of peer-based services later. This suggests that it may behoove PRS to cultivate client relationships early during contacts in order to reduce the diversity of needed service types later. Lastly, although dose at initial assessment related to some later outcomes (e.g., substance use, emergency and outpatient services), after applying a conservative alpha correction, dose was no longer associated with outcomes. Proposed mediators, self-efficacy and PRS relationship/helpfulness, also did not relate to outcomes. That said, PRS relationship was related to outpatient services (better relationship, less service use); however, with alpha correction, this relationship was no longer significant. No significant mediational effects were found in these analyses.

## Limitations

Although the overall sample was quite substantial (*N* = 1,544), only *n* = 412 (about 27%) of the sample was included in this study. That said, it is important to note that there were no substantive differences between those included in the study and those eliminated. This was because of the interest in assessing changes over time, and only participants who had data at both time points were examined. Examining only two time points also did not allow for within person differences to be assessed, given that such models require more waves of data to make those comparisons. Despite these limitations, this study was adequately powered. However, more research is needed to assess outcomes over a longer period.

Cannabis use in this study was aggregated with other substances (e.g., opiates, hallucinogens, etc.) into one single category (i.e., unregulated substance use), due to data for this construct not being normally distributed (i.e., highly kurtotic). Therefore, specific predictions could not be made about the impact of peer-based services on cannabis use. In addition, in the state where data were collected, medical use, decriminalization, and recreational use of cannabis changed over time. The questionnaire used for data collection did not specify whether cannabis was used for medical reasons or recreational use. Future research should attend to this distinction and better ascertain problematic use of cannabis.

The time between the first meeting with a PRS and the second meeting where the survey was completed could not be assessed in this study. Further, services provided were measured all together rather than separately (e.g., mental and substance use treatment vs. legal support vs. housing stability), which does not allow for a clearer assessment of what type of services may be more useful/beneficial for clients and their impact on outcomes. Future research would benefit from further separating the types of services provided to better inform prevention and treatment strategies. Despite the control of important extraneous variables in this study and application of rigorous statistical methods, there was no comparison group to assess impact of PRS vs no services or vs other services. Finally, future work may wish to measure and study other mediators according to a specific model of peer recovery. This might include focusing on measuring SAMHSA’s core competencies (e.g., helps to manage crises, values communication, links to services) of peer services.^14^

In spite of these limitations, strengths of this study include a large, well-powered data set, use of real-world data to evaluate services, conservative approach to family-wise error and alpha correction, examination of change over time, and rigorous analytic approach. This is important to the evaluation of peer services and to move the field forward.^8,9^

## Implications for Behavioral Health

In this study, mediation was not found; however, initial dose of peer services was associated with later better perceived relationship with and helpfulness of a PRS, while controlling for important covariates (e.g., sex). In addition, initial peer relationship/helpfulness was inversely associated with later dose of peer services. This suggests that when PRS put in initial effort, rewards might be reaped later (i.e., time now improves relations later, and good relations now can reduce service time later). It is possible that with a well-controlled study using validated instruments, associations might have been found for mediation. Nonetheless, this study contributes to the growing knowledge base on the implementation of peer services, and findings speak specifically to the relationship between services received and relationship with PRS. Findings in this study also support PRS’S continued integration in healthcare settings, including behavioral health, to maximize client outcomes. Future research should continue to explore these relationships.
